# Distinct moieties underlie biphasic H^+^ gating of connexin43 channels, producing a pH optimum for intercellular communication

**DOI:** 10.1096/fj.201700876R

**Published:** 2018-01-05

**Authors:** Carolina D. Garciarena, Akif Malik, Pawel Swietach, Alonso P. Moreno, Richard D. Vaughan-Jones

**Affiliations:** * Department of Physiology, Anatomy and Genetics, Burdon Sanderson Cardiac Science Centre, University of Oxford, Oxford, United Kingdom;; † Irish Centre for Vascular Biology, Royal College of Surgeons in Ireland, Dublin, Ireland;; ‡ Nora Eccles Harrison Cardiovascular Research and Training Institute, University of Utah, Salt Lake City, Utah, USA

**Keywords:** gap junctions, acid–base, heart, brain, electrical synapse

## Abstract

Most mammalian cells can intercommunicate *via* connexin-assembled, gap-junctional channels. To regulate signal transmission, connexin (Cx) channel permeability must respond dynamically to physiological and pathophysiological stimuli. One key stimulus is intracellular pH (pH_i_), which is modulated by a tissue’s metabolic and perfusion status. Our understanding of the molecular mechanism of H^+^ gating of Cx43 channels—the major isoform in the heart and brain—is incomplete. To interrogate the effects of acidic and alkaline pH_i_ on Cx43 channels, we combined voltage-clamp electrophysiology with pH_i_ imaging and photolytic H^+^ uncaging, performed over a range of pH_i_ values. We demonstrate that Cx43 channels expressed in HeLa or N2a cell pairs are gated biphasically by pH_i_ *via* a process that consists of activation by H^+^ ions at alkaline pH_i_ and inhibition at more acidic pH_i_. For Cx43 channel–mediated solute/ion transmission, the ensemble of these effects produces a pH_i_ optimum, near resting pH_i_. By using Cx43 mutants, we demonstrate that alkaline gating involves cysteine residues of the C terminus and is independent of motifs previously implicated in acidic gating. Thus, we present a molecular mechanism by which cytoplasmic acid–base chemistry fine tunes intercellular communication and establishes conditions for the optimal transmission of solutes and signals in tissues, such as the heart and brain.—Garciarena, C. D., Malik, A., Swietach, P., Moreno, A. P., Vaughan-Jones, R. D. Distinct moieties underlie biphasic H^+^ gating of connexin43 channels, producing a pH optimum for intercellular communication.

Cells of most human tissues—with the notable exception of blood cells and skeletal muscle cells—are electrically and metabolically coupled by means of gap junctional channels, assembled from connexin (Cx) proteins. The hexameric channels permit cell-to-cell solute and ion flow. This function plays a critical signaling role (1) that is particularly important for the spread of electric current in excitable tissues. The biological importance of gap junctional communication necessitates a means of regulating junctional permeability and conductance. Acute Cx channel regulation is typically exercised *via* post-translational modifications and may involve cellular metabolites and/or electrophysiologic maneuvers. Moreover, aberrant forms of Cx channel regulation have been implicated in pathologic states (2, 3), such as cardiac arrhythmias.

Among solutes that permeate Cx-assembled channels are H^+^ ions, the end products of metabolism. H^+^ ions are produced at a rate that reflects the tissue’s metabolic activity. They can feedback potently on cellular function *via* an array of protonation reactions with proteins. Essentially, all cell types are equipped with a molecular apparatus for maintaining favorable intracellular pH (pH_i_). Excess acid is commonly transferred from cells to the nearest functional blood capillary (4) *via* membrane transport proteins, such as H^+^-monocarboxylate transporters and Na^+^/H^+^ exchangers (NHEs). In addition, permeation of H^+^ ions through gap junctions allows pH_i_ to equilibrate spatially among cells, such as those of the working myocardium. Channel-facilitated H^+^ dissipation reduces the spatial heterogeneity of pH_i_, thereby helping to unify tissue-level function, such as myocardial contractility. In contrast, some clinical conditions—for example, myocardial ischemia—can trigger abnormally large decreases of tissue pH_i_; permitting a large and localized intracellular acid load to spread into surrounding tissue would risk inflicting undue damage on cells that are co-opted to share the pH_i_ disturbance. Instead, gap junctional channels tend to close by sensing low pH_i_.

A 1980s report first described an inhibitory effect of intracellular acidification on cell-to-cell coupling (5, 6). Subsequent expression studies on Cx43 channels have linked this to an inhibition by H^+^ ions, which relies on an interaction between the cytoplasmic C terminus of the Cx43 protein (residues 261–300 and 374–382) (7, 8) with its intracellular loop (a protonatable histidine residue) (9). Moreover, these domains are influenced by phosphorylation (10) and interactions with the cytoskeleton (11), which allows for additional fine tuning of Cx43 channel pH_i_ sensitivity. More recently, an additional pH_i_ control of gap junctional conductance and permeability has been described. Inhibition of electrical and solute coupling between mammalian ventricular myocytes—where Cx43 is the dominantly expressed gap junctional isoform—has been demonstrated at both low and high pH_i_. Ventricular coupling is thus modulated by pH_i_ in a biphasic manner, with peak conductance attained at pH_i_ ∼6.9, which is mildly acidic relative to normal resting pH_i_ (12). The molecular structures that underpin gap junctional block at high pH_i_ are currently unknown.

Here, by using heterologously expressed Cx43 channels, we confirm that alkaline—that is, high—pH_i_ reversibly and robustly reduces gap junctional communication, probed electrophysiologically and from measurements of cell-to-cell H^+^ ion permeation down a photolytically evoked gradient of [H^+^]_i_. Furthermore, by using mutants of Cx43, we show that the C terminus of Cx43 is involved in alkaline gating and that this process is independent of the molecular apparatus responsible for channel closure at acidic—that is, low—pH_i_. We present an updated model of the mechanism of biphasic gating of Cx43 channels by H^+^ ions. Our model explains the phenomenon of optimal Cx43 channel permeability in terms of the ensemble of inhibitory and activatory effects of H^+^ ions operating over distinct pH_i_ ranges.

## MATERIALS AND METHODS

### Cx43 expression and cell culture

HeLa (CRM-CCL2; American Type Culture Collection, Manassas, VA, USA) and N2a (CCL-131; American Type Culture Collection) cells were transfected with cDNA for rat Cx43 in pcDNA3.1 vectors (13). Truncated Cx43 (Cx43m257HT) was generated by introducing a stop codon at residue 258 into the cloning site of the bicystronic vector, pIRES2 (Clontech, Mountain View, CA, USA) that provides resistance to Geneticin (G418; Mutagenesis Kit; Thermo Fisher Scientific, Waltham, MA, USA) and includes a histidine tag (His)_6_. Mutants with ≥1 cysteine-to-alanine mutations at the C tail were generated by PCR using specific primers (Supplemental Fig. 1), and the gene was introduced into a pcDNA vector with G418 resistance. Mutant constructs that have all serine residues of the PKC epitope (356–389) substituted to alanine were prepared by PCR using specific primers, similar to those shown in Supplemental Fig. 1. This phospho-null mutant gene was introduced into a pcDNA vector with hygromycin resistance. Transfected cells were selected with 150 μg/ml hygromycin B or 800 μg/ml G418. Cells were grown in high-glucose DMEM that was supplemented with 10% fetal bovine serum, penicillin, streptomycin, glutamine, and normocin (Thermo Fisher Scientific) at 37°C and 5% CO_2_. Cell pairs were obtained by allowing for 1 division cycle (8- to 24-h culture for HeLa; 72-h culture for N2a).

### Western blot and immunofluorescence

Cell lysates were prepared from confluent monolayers with RIPA buffer that contained protease/phosphatase inhibitors (Roche, Basel, Switzerland). After centrifugation at 5000 *g*, protein was subject to 12% SDS-PAGE, transferred to PVDF membranes, and probed with primary Abs. Signal was detected by chemiluminescence that was obtained from secondary horseradish peroxidase–conjugated anti-rabbit, -goat, or -mouse Abs (Thermo Fisher Scientific) and ECL (GE Healthcare, Pittsburgh, PA, USA). For immunofluorescence, cells that were grown on coverslips were fixed with 4% paraformaldehyde for 10 min, permeabilized with 0.1% Triton X-100 for 20 min, blocked with 1% bovine serum albumin (1 h), incubated with primary Abs (1:50; 1 h), and incubated with Alexa Fluor-488/-647–conjugated secondary Abs (1:200; 1 h). Primary Abs were against Cx43 (rabbit polyclonal; EMD Millipore, Billerica, MA, USA), actin (goat polyclonal; Santa Cruz Biotechnology, Dallas, TX, USA), His-Tag (mouse monoclonal; EMD Millipore), and NHE1 (mouse monoclonal; BD Biosciences, San Jose, CA, USA). Coverslips were mounted on slides by using DAPI-containing mounting medium (ProLong; Thermo Fisher Scientific). Images were acquired with an inverted laser-scanning microscope (SP5; Leica Microsystems, Buffalo Grove, IL, USA); excitation/emission wavelengths were 351 nm/400–440 nm (DAPI), 488 nm/500–600 nm (Alexa Fluor-488), and 633 nm/>650 nm (Alexa Fluor-647).

### Electrophysiology

Dual whole-cell voltage clamp was performed on cell pairs by using 3- to 5-MΩ pipettes that were filled with intracellular solution (in mM: 143 CsCl, 10 NaCl, 5.5 glucose, 1 MgCl_2_, 3 HEPES, pH 7.1). Measurements were performed at room temperature. A continuous protocol of 10-mV/10-ms hyperpolarizing pulses was applied sequentially to each cell of the pair at 0.5 Hz by using an analog stimulator (Winston Electronics, St. Louis, MO, USA). For longer-lasting experiments—namely, those that involve studies of Cys-to-Ala mutants—cell triplets were used for double voltage-clamp experiments as these produced more stable recording over many minutes compared with cell pairs. Two voltage-clamp amplifiers (Warner 201A) were used to determine *trans*-junctional current (*I*_j_). Junctional conductance (*G*_j_) was calculated by dividing *I*_j_ by the applied voltage. Signals were acquired at 5 kHz and filtered at 1 kHz. Experiments were conducted at room temperature and results were normalized to the value at the start of the experiment. Single channel-unitary currents were obtained after adding 2 mM halothane to the bathing solution to reduce channel open probability, which makes it possible to record single-channel events from cell pairs. This is critical because junctional plaques will inevitably contain many Cx channels, which would be a confounding factor in measuring single-channel properties. A driving force of 30–60 mV was applied to calculate channel-unitary conductance. Current signals were filtered at 100 Hz. pH_i_ was monitored by using an epifluorescence system coupled to a Nikon inverted microscope (Nikon, Tokyo, Japan) using 5-(and-6)-carboxy seminaphtharhodafluor-1 (SNARF-1) (14) loaded into cells as their acetoxymethyl ester (AM; 10 µM) for 10 min (Molecular Probes, Eugene, OR, USA). Excitation at 515 nm was provided by a mercury-arc lamp, and fluorescence at 640 ± 20 and 580 ± 20 nm was collected by 2 photomultiplier tubes that were equipped with band-pass filters. The fluorescence emission ratio (640/580) was digitized at 5–10 kHz (Digidata 1322A; Molecular Devices, Sunnyvale, CA, USA). Emission ratio was calibrated as previously described (14).

### pH_i_ imaging, buffering capacity, and H^+^ flux measurement

HeLa or N2a cells—AM loaded with 5-(and-6)-carboxy SNARF-1 (10 µM for 10 min)—were imaged confocally (514-nm excitation; emission at 630–650 and 580–600 nm) and calibrated as previously described (14). Hepes-buffered superfusates contained (mM): 20 HEPES, 135 NaCl, 4.5 KCl, 1 CaCl_2_, 1 MgCl_2_, 11 glucose; pH was adjusted to 7.4 with 4 M NaOH at 37°C. CO_2_/HCO_3_^−^-buffered superfusates contained (mM): 22 NaHCO_3_, 125 NaCl, 4.5 KCl, 1 CaCl_2_, 1 MgCl_2_, 11 glucose; pH was adjusted to 7.4 by bubbling with 5% CO_2_–95% air at 37°C. H^+^ fluxes were calculated from cell-averaged pH_i_ changes (*d*pH_i_/*dt*) as *J*_H_ = −β_tot_ × *d*pH_i_/*dt*, where β_tot_ is total intracellular buffering capacity, the sum of CO_2_/HCO_3_^−^ buffering (β_CO2_) and intrinsic buffering (β_int_) components. β_int_ was estimated from pH_i_ changes induced by the stepwise removal of extracellular NH_4_Cl. β_CO2_ was calculated as 2.3 × [HCO_3_^−^]_i_ (14).

### H^+^ uncaging and apparent junctional H^+^-permeability measurements

Superfusates contained 1 mM 2-nitrobenzaldehyde, a photo-labile, membrane-permeable H^+^ donor substance (15). Uncaging was performed in a 5 × 5-μm region of interest in one cell of a pair—referred to as cell_1_—by 351 nm UV-light—3 flashes—every 3.6 s. The rate of uncaging was estimated from the product of the change in pH_i_, evoked by a triple-flash event, and buffering capacity (Supplemental Fig. 4). Per uncaging event, 0.3 mM H^+^ ions are released (equivalent to 5 mM H^+^/min). Acid extrusion by the membrane transporters, NHE1 and Na^+^-HCO_3_^−^, cotransport was inhibited by 30 μM dimethyl amiloride and 150 μM 4,4′-diisothiocyano-2,2′-stilbenedisulfonic acid included in superfusates. Apparent junctional H^+^-permeability (*P*_H_^app^) was calculated from the initial time course of H^+^ permeation—consisting of 40 s of baseline and the first 54 s of H^+^ uncaging—by fitting a diffusion permeation algorithm (16).

### pH_i_ manipulation

pH_i_ was manipulated by superfusion with solutions in which weak acid or base osmotically replaced NaCl and extracellular pH was kept constant at 7.4, unless indicated otherwise. Intracellular acid and alkaline loads were achieved with 40–160 mM Na^+^ acetate and 10–40 mM trimethylamine (TMA), respectively.

### Ca^2+^ imaging

[Ca^2+^]_i_ was measured in cell pairs that were AM-loaded with Fluo3 (10 μM; Molecular Probes), excited at 488 nm, and with emitted fluorescence collected at >505 nm. After subtracting background fluorescence, Fluo3 time course signal was normalized to baseline (*F*/*F*_0_).

### Statistics

Summarized electrophysiologic results of changes in *G*_j_ are expressed as means ± sd, whereas summarized permeability results are expressed as means ± sem. A paired Student’s *t* test was used to test the significance between results obtained with each cell pair serving as its own control. An unpaired *t* test was used to test significance between results that were obtained on different cells pairs. Values of *P* < 0.05 were considered significant.

## RESULTS

### Cells transfected with the Cx43 gene become electrically uncoupled at low and high pH_i_

**Figure 1.**
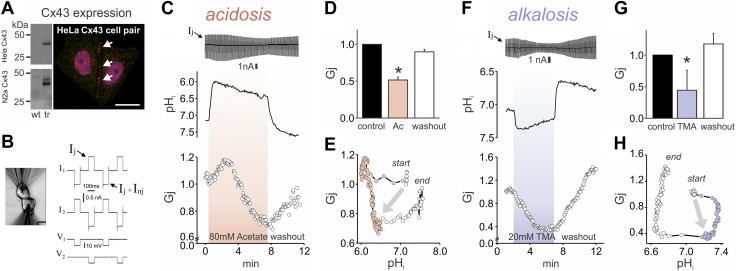
Electrical uncoupling of Cx43 cell pairs at low and high pH_i_. *A*) Cx43 protein in HeLa and N2a cells confirmed by Western blot analysis and immunostaining. Wild-type HeLa and N2a lack Cx43 endogenous. Multiple bands correspond to Cx43 phosphorylation states. Cx43 localizes at the interface between cells (white arrows). Cells were stained by using anti-Cx43 Ab (red). Nuclei were labeled with DAPI (purple). Scale bar, 25 µm. *B*) Junctional conductance (*G*_j_) assessed by double whole-cell voltage clamp on cell pairs. During alternating voltage pulses, junctional currents (*I*_j_) are a readout of cell-to-cell coupling and *I*_j_ + *I*_nj_ represent the junctional plus transmembrane currents in the stimulated cell. *C*) Example experiment showing effect of superfusion with 80 mM Na_+_ acetate. Junctional currents (increase indicates stronger coupling; top). Time course of pH_i_ measured by using SNARF-1, showing rapid acidification, secondary partial recovery, and a rebound alkalinization upon washout (middle). Low pH_i_ reduced *G*_j_ rapidly (bottom). Note the small initial transient increase in *G*_j_, followed by a fast decrease that reached ∼70% of initial value. *D*) Average data (mean ± sd) for *G*_j_ at baseline conditions, reduction after 3 min of acetate, and recovery after 5 min washout (*n* = 5, HeLa and N2a cell pairs). *E*) Representative plot of pH_i_ *vs.* *G*_j_. After the start position (all white circles), there is a robust reduction in pH_i_, whereas *G*_j_ remained constant. This is followed by an increase in *G*_j_, then a decrease. Arrow indicates the net change in *G*_j_ and pH_i_ during the protocol. During washout (all white circles), pH_i_ and *G*_j_ recover toward initial levels. *F*) Example experiment showing effect of superfusion with 20 mM TMA. *I*_j_ (top). Time course of pH_i_ showing rapid alkalinization, secondary partial recovery, and a rebound acidification upon washout (middle). Alkaline pH_i_ reduces *G*_j_ toward ∼50% of the initial *G*_j_ value (bottom). *G*) Average (mean ± sd) *G*_j_ at baseline conditions, after 3 min perfusion of TMA, and recovery after 5 min of washout (*n* = 5 N2a cell pairs). *H*) Representative plot of pH_i_ *vs.* *G*_j_ plot. Arrow indicates net change in *G*_j_ and pH_i_ near the steady state. *P < 0.05.

*Gja1*, the gene that codes for rat Cx43 protein, was stably transfected into HeLa and N2a cells. Cx43 protein was confirmed by Western blot analysis and immunofluorescence (**Fig. 1*A***). Cx43-positive plaques formed at the interface between cells. Junctional conductance (*G*_j_)—calculated from double whole-cell voltage-clamp pulses (Fig. 1*B*)—was abrogated by the addition of the gap junctional blocker, β-glycyrrhetinic acid (βGA; 60 µM). These observations are attributable to the transfected gene because wild-type HeLa and N2a cells lack endogenous Cx43 (Fig. 1*A*) and have no detectable junctional conductance.

Effects of intracellular acidosis were studied in HeLa and N2a cells that were transfected with Cx43. Superfusion with 80 mM Na^+^ acetate produced a prompt acidification (fall of pH_i_), followed by a small degree of recovery (Fig. 1*C*, middle) that was attributable to acid extrusion by transporters, such as NHE (flux analysis in Supplemental Fig. 2). Intracellular acidification first produced a small transient rise in *G*_j_, followed by a delayed and slower decrease that reached a nadir of ∼70% of the control value (Fig. 1*C*, bottom). Withdrawal of acetate alkalinized pH_i_ and evoked a rise in *G*_j_, which was indicative of a reversible block (Fig. 1*C*, bottom). These *G*_j_ changes are additionally quantified in Fig. 1*D*. Of note, the *G*_j_ response lagged behind the pH_i_ change by tens of seconds, which indicates a time dependence of acid-evoked block and its subsequent recovery (illustrated as a hysteresis loop in Fig. 1*E*).

The effect of raising pH_i_ with 20 mM TMA was determined in separate experiments on Cx43-transfected N2a and HeLa cells. Raising pH_i_ produced a monophasic, albeit delayed, fall in *G*_j_ to ∼40% of control conductance (Fig. 1*F*, bottom). As with the response to acidosis, the effect of raising pH_i_ was reversible (Fig. 1*F*) and is additionally quantified in Fig. 1*G*. The *G*_j_ response, again, was delayed relative to the rapidly imposed pH_i_ maneuvers, as illustrated in the hysteresis plot shown in Fig. 1*H*. Overall, changes of pH_i_ in either direction from the resting value of ∼7.1 reduced junctional conductance, but with a significant time delay. One interpretation of this result would be an activatory effect on *G*_j_ of Cx43 channel protonation over the alkaline pH_i_ range, but an inhibitory effect of protonation over a more acidic range, with a net transition that occurred near pH_i_ ∼6.9—the latter would explain the rise, then fall of *G*_j_ upon acidification from pH_i_ ∼7.1 (see Fig. 1*C*).

**Figure 2.**
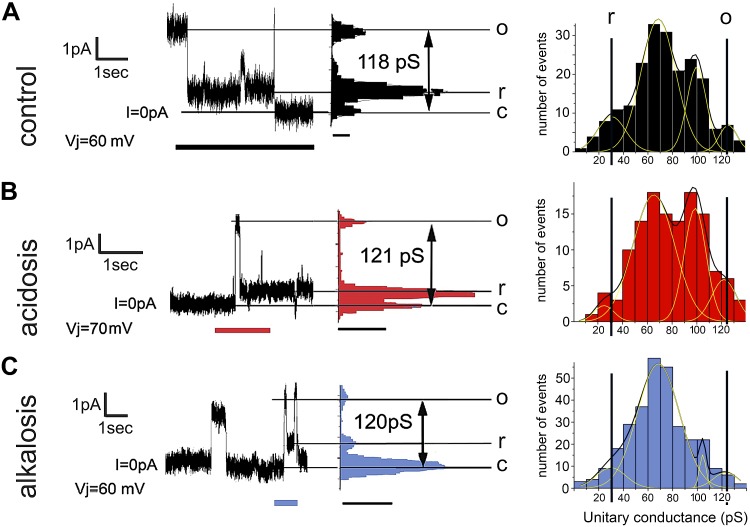
Effect of pH_i_ on single-channel properties. *A*) Single-channel current recordings from Cx43-transfected HeLa cells measured in 2 mM halothane. Example time course shows rapid transitions from open to residual or closed states. Point-to-point histogram (middle*)* shows the fully open conductance (o) of 118 pS and a residual conductance (r) of 30 pS. All-event histogram (right) indicates the number of transitions between states. *B*). Single-channel recordings during the application of 80 mM acetate. Point-to-point histogram (middle) shows o = 121 pS and r = 30 pS. *C*) Single-channel recordings during the application of 20 mM TMA. Point-to-point histogram (middle) shows o = 120 pS and r = 30 pS. Unitary conductance measured in homotypic channels is not significantly different from previously reported values (120 pS). All-event histograms include peaks from transitions between fully open minus residual state (120−30 = 90 pS), and fully open channel transitions of phosphorylated states (60 pS).

To explore the mechanism of pH_i_ sensitivity of *G*_j_, Cx43 single-channel conductance was recorded in HeLa cells—in the presence of 2 mM halothane, which rapidly blocks the majority of channels (**Fig. 2*A***). Cx43 channels typically exhibit one maximal conductance state that corresponds to the dephosphorylated Ser368 open state (o, ∼120 pS), and a residual conductance state (r, ∼30 pS) observed upon application of a *trans*-junctional voltage (13). In addition, a number of events are observed near 60 pS, considered to be the unitary conductance of phosphorylated connexin. Acidosis—attained with 80 mM Na^+^ acetate (Fig. 2*B*) or alkalosis—attained with 20 mM TMA (Fig. 2*C*)—produced no significant change in the amplitude of single-channel conductance (open state, o), with a small shift in the unitary conductance event distribution during alkalosis. In conclusion, changes in the maximal conductance of single Cx43 channels are unlikely to explain the pH_i_ responses described in Fig. 1.

### Probing Cx43 channel permeability from measurements of H^+^ ion permeation between cells

**Figure 3.**
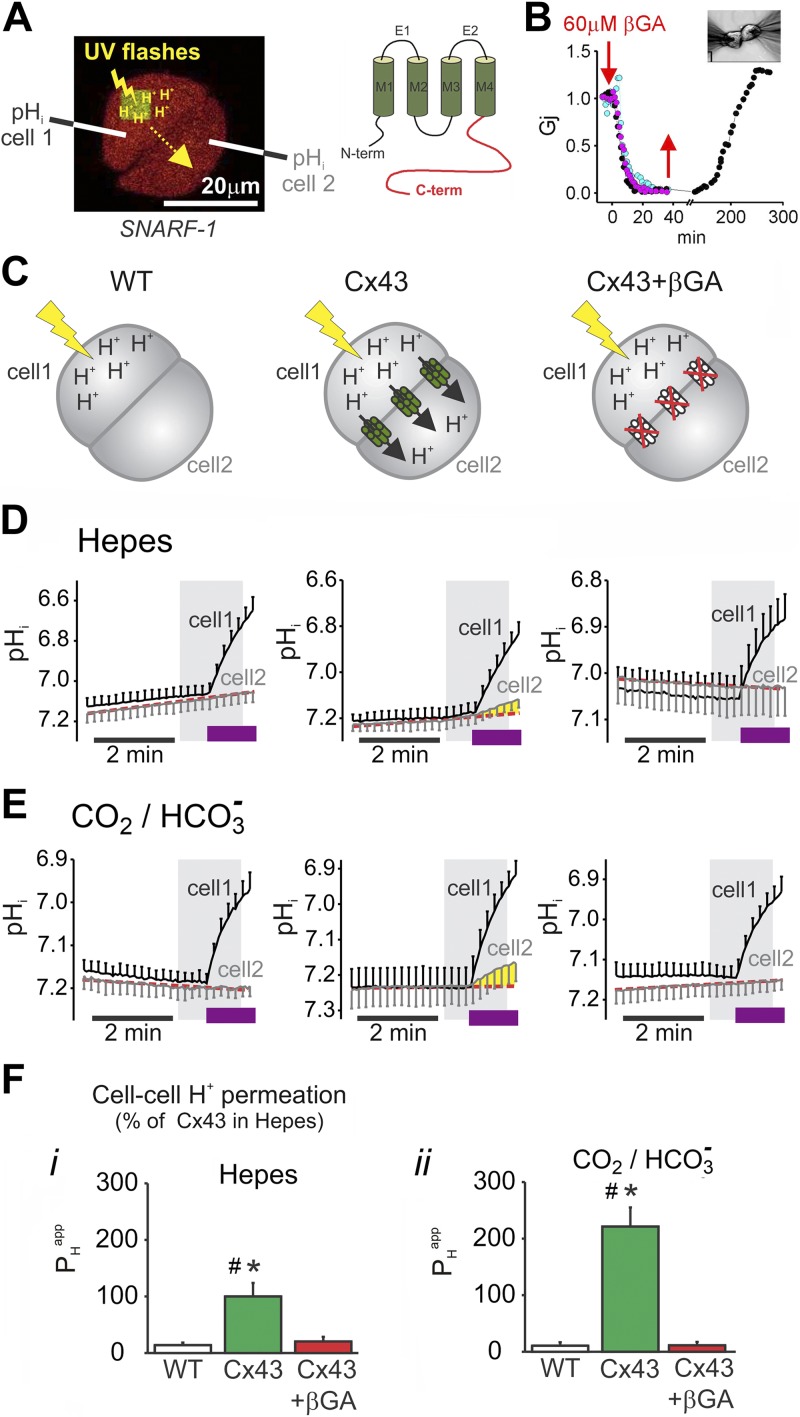
Cx43-mediated junctional H^+^ ion permeation. *A*) Photolytic H^+^ uncaging (triple flash of UV light/3.6 s) in 5 × 5-µm region of interest from 2-nitrobenzaldehyde (1 mM) in one cell of Cx43-transfected N2a cell pair produces a local acidification [confocally imaged 5-(and-6)-carboxy SNARF-1 fluorescence] that spreads to the distal cell *via* Cx43 channels, if active (left). Cartoon representation of wild-type Cx43 protein (right). *B*) N2a cells expressing wild-type Cx43 become uncoupled by 60 μM βGA (colored traces represent individual recordings). *C*) Schematic representation of the different coupling states from which permeability data are obtained (wild-type, Cx43 expressor, and Cx43 expressor inhibited with βGA). *D*) Cell-to-cell H^+^ ion permeation measured in Hepes-buffered superfusates in N2a cells (Cx43-negative). To highlight pH_i_ changes in the recipient cell (cell-2), the dashed line extrapolates baseline. Substantial acidification of cell-2 is observed in Cx43 transfectants and is βGA sensitive. *E*) Cell-to-cell H^+^ ion permeation measured in CO_2_/HCO_3_^−^ buffered superfusates. For panels *D*, *E*, the shaded regions correspond to the period we used to fit data using the mathematical models. Purple bands correspond to the uncaging period (flash photolysis). *F*) Summary of *P*_H_^app^ data. All H^+^ uncaging experiments were performed in the presence of 30 μM dimethylamiloride to inhibit acid extrusion by the NHE. Experiments in CO_2_/HCO_3_^−^ buffer were also performed in the presence of 150 μm 4,4′-diisothiocyano-2,2′-stilbenedisulfonic acid. ^#^Significantly different from 0 (*P* < 0.05). *P < 0.001.

Cx43 channel gating was also interrogated from measurements of cell-to-cell permeability to H^+^ ions. To drive a net flux of H^+^ ions through gap junctions, one cell of a pair was acidified by a series of photolytic uncaging reactions that involved the membrane-permeable H^+^ donor, 2-nitrobenzaldehyde, which was dissolved in superfusates at 1 mM (15) (**Fig. 3*A***, left). The diffusive spread of uncaged H^+^ ions was then monitored by imaging pH_i_ in the cell pair at intervals between local uncaging events. To obtain an estimate of *P*_H_^app^, [H^+^]_i_ time courses in the source and recipient cells were best fitted to a permeation algorithm. It is well established (12, 17) that H^+^ ions permeate through connexin channels aboard small, mobile buffer molecules; therefore, calculated *P*_H_^app^ is a dual function of the channels’ permeability state and the availability of permeant buffers for the shuttling of H^+^ ions through gap junctions.

*P*_H_^app^ measurements were first performed on wild-type or Cx43-transfected N2a cells. Transfected N2a cells had βGA-inhibitable junctional conduction (Fig. 3*B*), which confirmed the functional expression of Cx43. Uncaging experiments were performed on cells that were superfused with CO_2_/HCO_3_^−^-free (Hepes) superfusates to measure H^+^ ion permeation aboard intrinsic mobile buffers (Fig. 3*C*). Acid extrusion by membrane transporters was blocked with 30 µM dimethylamiloride, an NHE1 inhibitor. In agreement with electrophysiologic recordings, wild-type cells displayed no evidence for H^+^ ion permeation between cells (Fig. 3*D*, left), but significant transmission was observed between Cx43-transfected cells (Fig. 3*D*, middle), which was inhibitable with βGA (Fig. 3*D*, right). *P*_H_^app^ data are summarized in Fig. 3*F**i*. In a second series of experiments, *P*_H_^app^ was probed in the presence of 5% CO_2_/22 mM HCO_3_^−^ buffer, which introduced additional mobile buffering into the cytoplasm. Acid extrusion was blocked pharmacologically with 30 µM dimethylamiloride and the HCO_3_^−^ transport inhibitor, 4,4′-diisothiocyano-2,2′-stilbenedisulfonic acid (150 µM; Supplemental Fig. 2). *P*_H_^app^ was 2-fold higher under this buffering regime (Fig. 3*E, F**ii*), which was consistent with the junctional permeation of additional mobile buffers provided by cytoplasmic CO_2_/HCO_3_^−^ (15). Paracellular diffusion of CO_2_ was ruled out on the basis of the absence of cell-to-cell H^+^ ion transmission in wild-type cells and the complete block of transmission by βGA in Cx43-expressing cells (Fig. 3*F**ii*). Similar results were obtained with HeLa cells (Supplemental Fig. 3). H^+^ ion permeation time courses were analyzed in terms of junctional H^+^ flux (Supplemental Fig. 4). For an uncaging rate of ∼5 mM H^+^/min, at pH_i_ ∼6.95, the H^+^ ion flux through Cx43 channels was 1.0 mM H^+^/min in Hepes buffer and 1.7 mM H^+^/min in CO_2_/HCO_3_^−^ buffer (Supplemental Fig. 5).

### Biphasic regulation of Cx43 channels’ H^+^ ion permeability by cytoplasmic H^+^ ions

**Figure 4.**
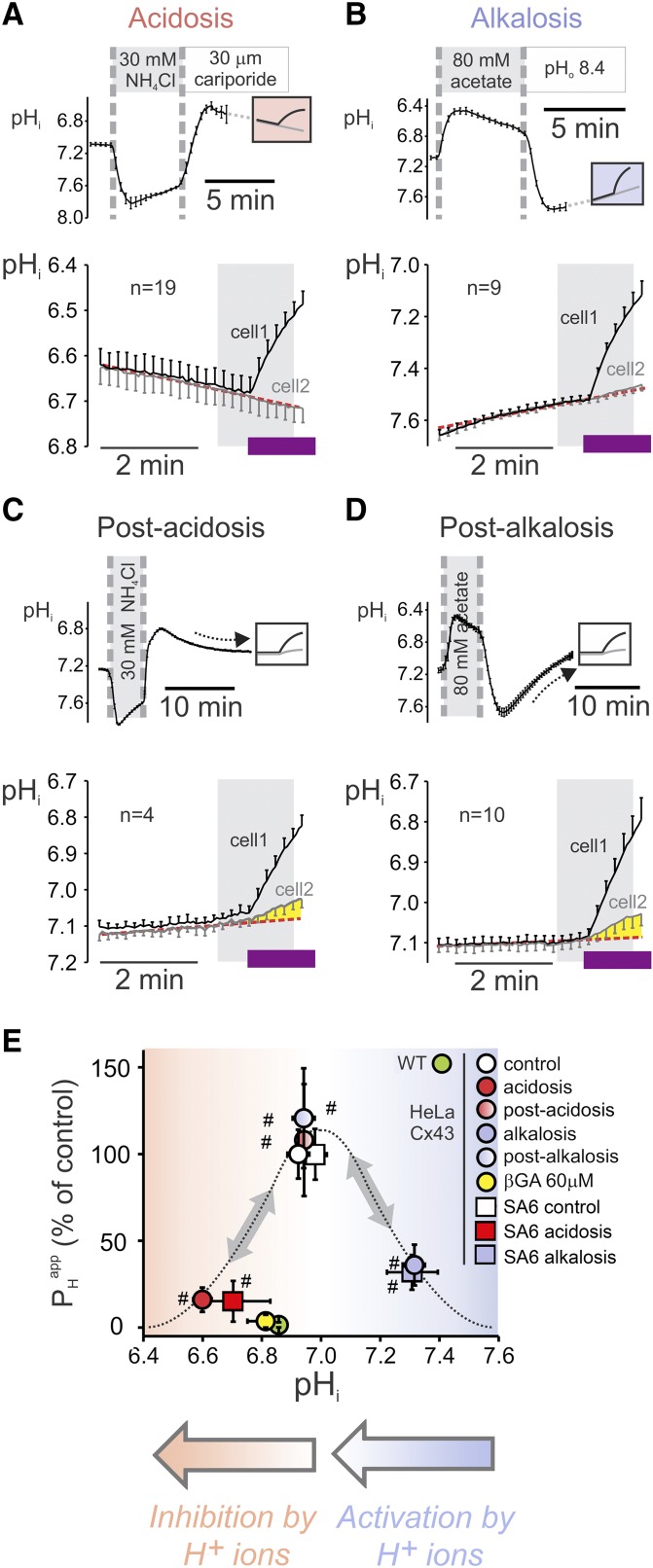
Reversibility of pH_i_ gating. *A*) Resting pH_i_ in Cx43-transfected N2a cell pairs was preadjusted to acidic before H^+^ uncaging by 30 mM NH_4_Cl prepulse (returned to normal Tyrode that contained 30 µM dimethylamiloride to inhibit pH_i_ recovery). pH_i_ time course (bottom) for estimating junctional H^+^ ion permeability (*P*_H_^app^) showing reduced transmission. *B*) Resting pH_i_ preadjusted to alkaline before H^+^ uncaging by 80 mM Na^+^ acetate prepulse (returned to normal Tyrode at pH 8.4 to slow pH_i_ recovery). pH_i_ time course (bottom) for estimating *P*_H_^app^ shows significant reduction in transmission. *C*) Acid block was reversed after allowing 10 min of recovery from acidic pH_i_. *D*) Alkali block was reversed after allowing 10 min of recovery from alkaline pH_i_. Shaded regions correspond to the period we used to fit data using mathematical models. Purple bands correspond to the uncaging period (flash photolysis). *E*) Summary of reversibility results were fitted to a bell curve. Added to this data are the results obtained from acidosis and alkalosis experiments performed by using Cx43 mutants where all serine residues from the PKC epitope at the end of the C tail have been substituted by alanine residues (SA6). Activation and inhibition by H^+^ ions are highlighted by gray arrows. ^#^Significantly different from 0.

In the next series of experiments, Cx43 channel activity was characterized over a range of pH_i_ values by using the protocol for measuring *P*_H_^app^ in Cx43-transfected HeLa cells. Experiments were performed in Hepes-buffered superfusates to ensure that only the intrinsic mobile buffers are responsible for shuttling H^+^ ions between cells. Use of weak acids or bases to manipulate pH_i_—as was done in electrophysiologicl recordings (Fig. 1)—was now avoided to eliminate the possibility of exogenous weak acid–base-facilitated H^+^ ion permeation, which may, in principle, supplement the effect of intrinsic buffers. Instead, pH_i_ was manipulated by means of a prior solution maneuver that shifted pH_i_ to a new level (prepulse). Cells were superfused with a weak acid (or base) for 5 min, then returned to normal solution. pH_i_ then rebounded to an alkaline (or acidic) level. At the displaced level of pH_i_, measurements of *P*_H_^app^ could be made with no interference from the exogenous weak acid–base because, at that point, the exogenous substance had been washed away. Superfusion with 30 mM NH_4_Cl, followed by washout in normal Tyrode-containing 30 µM dimethylamiloride (NHE1 inhibitor), induced a sustained intracellular acid load (**Fig. 4*A***), whereas superfusion with 80 mM acetate, followed by washout with normal Tyrode at pH 8.4, induced an intracellular alkali load (Fig. 4*B*). (Note: alkaline superfusates block any membrane transporters that might otherwise acidify cytoplasm.) In calculating *P*_H_^app^, the slow recovery of pH_i_ from an acid or alkali load was corrected by extrapolating the trend line that was measured before photolysis. Cell-to-cell H^+^ ion transmission was reduced substantially at acidic and alkaline pH_i_, which confirmed biphasic pH_i_ gating (Fig. 4*A, B*).

To assess whether the inhibitory effect of acute acidosis and alkalosis on *P*_H_^app^ was reversible, repeat measurements were made once pH_i_ had stabilized nearer to resting levels (N2A cells). As expected from a reversible H^+^-dependent block, *P*_H_^app^ was restored to control levels upon pH_i_ recovery from an acidic (Fig. 4*C*) or alkaline load (Fig. 4*D*). In summary, measurements of the pH_i_ dependence of *P*_H_^app^ confirm the observations—made using an electrophysiologic approach (Fig. 1)—that Cx43 channels are gated by low and high pH_i_ within minutes and that this process is fully reversible. A plot of *P*_H_^app^ *vs.* pH_i_—*x*-coordinate taken as the pH_i_ in the source cell averaged over a 1-min period of uncaging—demonstrates that acidification from 6.9 to 6.6 decreased Cx43 *P*_H_^app^ by 80%, whereas alkalinization from 6.9 to 7.3 caused *P*_H_^app^ to decrease by 60% (Fig. 4*E*).

To investigate whether changes in the phosphorylation of serine, particularly residue Ser368, underpin the decrease in *G*_j_ at acidic or alkaline pH_i_, additional experiments were performed on Cx43 mutants [Cx43(S-A)_6_] that had all serine residues of the PKC epitope, including Ser368, substituted by alanine, *i.e.*, a phospho-null variant. This mutant, expressed in HeLa cells, retained the response to acetate and TMA, which argues against a role for phosphorylation changes in pH_i_ dependence (Fig. 4*E*).

At least part of the pH_i_ dependence of *P*_H_^app^ may be attributed to changes in the availability of mobile buffers—that is, the fraction of buffering capacity held on small molecules (Φ = β_mobile_/β_total_), such as CO_2_/HCO_3_^−^, phosphates, and dipeptides that shuttle H^+^ ions through Cx43 channels (17). Mathematically, *P*_H_^app^ can be deconvoluted as the product of Φ and *P*_mobile_ (pH-gated channel permeability to mobile buffers). In principle, both *P*_mobile_ and Φ can display pH dependence, but only the former relates to Cx43 channel activity. To confirm that the pH_i_ sensitivity of *P*_H_^app^ (Fig. 4*E*) reflects, at least in part, the H^+^ gating of Cx43 channels, it is necessary to rule out a biphasic pH_i_ sensitivity of Φ. This was evaluated by considering 2 models. In the first, β_mobile_ was assumed to be a constant fraction of β_total_—that is, Φ would take a pH-independent constant value (0 < Φ <1) and, consequently, the observed pH_i_ dependence of *P*_H_^app^ would purely be a phenomenon of the pH_i_ sensitivity of *P*_mobile_. In an alternative model, β_mobile_ and β_total_ are assumed to have opposite pH dependence—that is, Φ is not constant. In most cells, including N2a cells, the pH_i_ dependence of intrinsic β has a negative slope over the physiologic pH_i_ range [Supplemental Fig. 4; in N2a: β_total_ = 95−11 × pHi (millimolar)]. This negative relationship arises because fixed buffers, mainly proteins, which dominate the intrinsic buffer pool demonstrate peak buffering in the acidic range. In contrast, less abundant mobile buffers typically have a more alkaline pH_i_ optimum (18). Effectively, Φ would be a positive function of pH_i_, thereby eliminating the possibility that alkaline inhibition of *P*_H_^app^ is caused by a fall in Φ; however, the reduction in *P*_H_^app^ that was observed at low pH_i_ may still relate to a decrease in Φ. This, however, is not quantitatively consistent with experimental observations. A decrease in pH_i_ from 7.3 to 6.6 was associated with an 80% decrease in *P*_H_^app^, which cannot be explained by a decrease in Φ alone because buffering capacity is noncooperative and, therefore, not associated with a steep pH_i_ dependence. In summary, the biphasic shape of the pH_i_-*P*_H_^app^ relationship, at least in part, must be a result of H^+^ gating of Cx43 channels. Furthermore, the alkaline part is wholly attributable to an H^+^ gating of Cx43 channels.

### Biphasic H^+^ gating of Cx43 channels is not explained by pH-evoked Ca^2+^ signals

**Figure 5.**
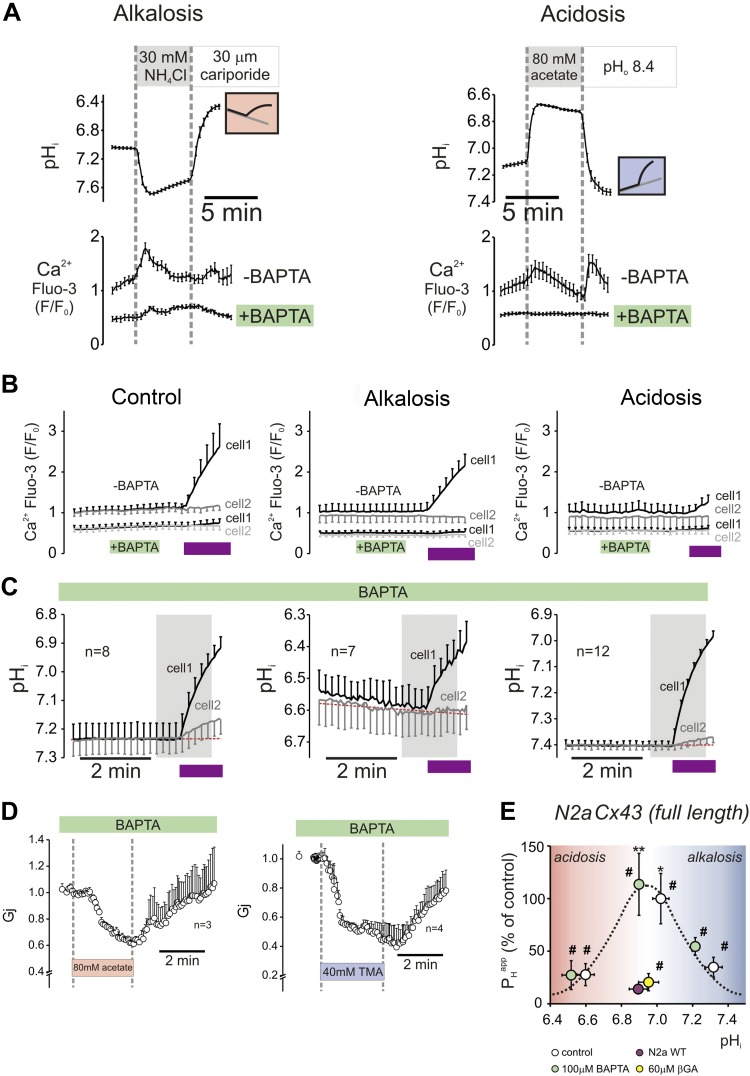
Ca^2+^ signals are not involved in the alkaline-gating mechanism. *A*) Prepulsing with 30 mM NH_4_Cl (left) or 80 mM acetate (right) normally increases [Ca^2+^] (Fluo3), but this is attenuated in cell pairs that are AM-loaded with 100 µM BAPTA. *B*) BAPTA also ablates Ca^2+^ response to H^+^ uncaging. *C*) BAPTA did not affect H^+^ ion permeability, nor its pH_i_ dependence. Shaded regions correspond to the period we used to fit data using mathematical models. Purple bands correspond to the uncaging period (flash photolysis). *D*) Inclusion of 5 mM BAPTA in the pipette solution during whole-cell voltage-clamp experiments had no effect on *G*_j_ response to 80 mM acetate or 40 mM TMA. Controls to this experiment (BAPTA-free pipettes) are shown in Fig. 1. *E*) Comparison of *P*_H_^app^ calculations between control (white circles) and BAPTA (green circles) experiments. Also included are results from nontransfected N2a cells (red circle) and Cx43-transfected cells that were treated with 60 μM βGA (yellow circle). ^#^Significantly different from 0.

Changes in pH_i_ can displace Ca^2+^ ions from intracellular buffers or subcellular compartments (19). As robust cytoplasmic Ca^2+^ signals are known to produce Cx43 channel closure (5), the acidic responses of *G*_j_ and *P*_H_^app^ may, in principle, involve an increase in [Ca^2+^] (20). To investigate whether the pH_i_ sensitivity of Cx43 function had an underlying Ca^2+^-dependent component, pH_i_ gating responses were measured in cells that were preloaded with the Ca^2+^ buffer, BAPTA (AM ester; 100 µM), which minimized any acidosis-evoked changes in [Ca^2+^] (Fluo3 fluorescence). Prepulsing with 30 mM NH_4_Cl or 80 mM Na^+^ acetate transiently increased Fluo3 fluorescence, but this response was ablated in cells that were preloaded with BAPTA (**Fig. 5*A***). The effect of BAPTA on the pH_i_ dependence of *P*_H_^app^ was investigated by using the photolytic protocol performed in CO_2_/HCO_3_^−^-free superfusates. H^+^ uncaging induced an increase in cytoplasmic [Ca^2+^] (Fig. 5*B*), but cells that were pretreated with BAPTA showed no acid-evoked [Ca^2+^] response, yet retained pH sensitivity of *P*_H_^app^ (Fig. 5*C, E*). As additional confirmation for the absence of a meaningful Ca^2+^-dependent component, the inclusion of 5 mM BAPTA in the pipette solution had no effect on *G*_j_ response to 80 mM Na^+^ acetate or 40 mM TMA (Fig. 5*D*).

### C tail of Cx43 is linked to the mechanism of alkaline gating of channel activity

**Figure 6.**
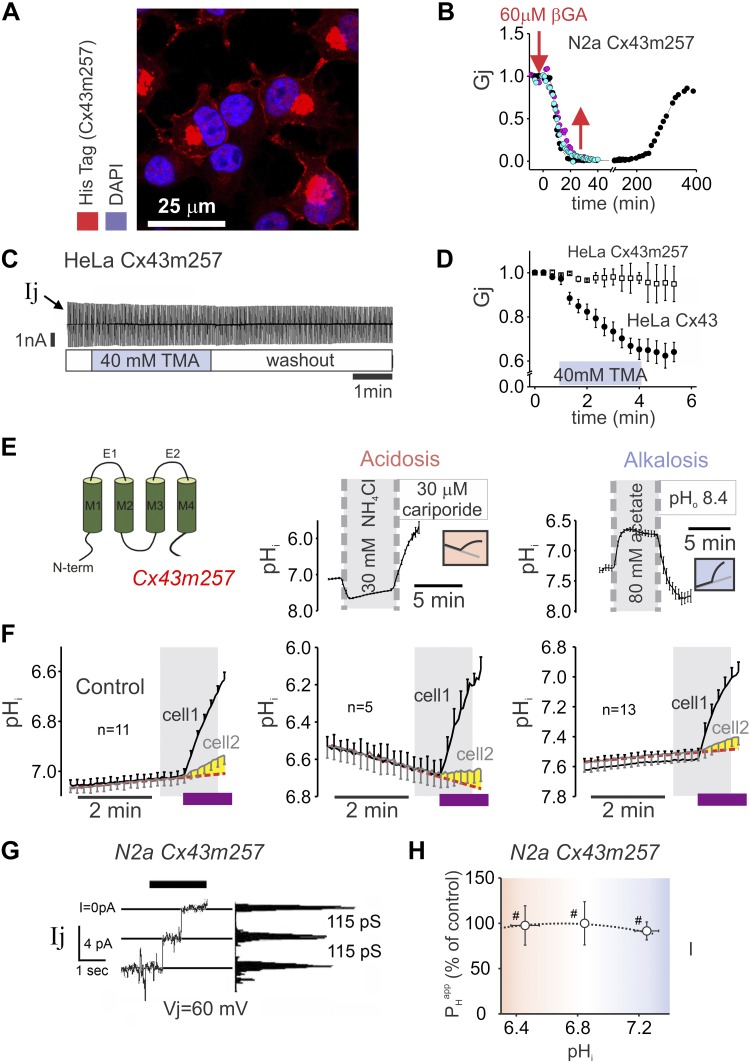
Role of Cx43 C-tail domain in pH_i_ gating. *A*) Confocal image showing the expression of truncated Cx43 (Cx43m257) tagged with a His-tag domain at the end of the C terminus (red). DAPI staining identified nuclei (blue). *B*) N2a cells expressing truncated Cx43 become uncoupled after the application of 60 μM βGA (2 separate experiments in red and blue). The effect was reversible (black). *C*) Junctional current (*I*_j_) does not change during 40 mM TMA perfusion during the application of voltage pulses shown in Fig. 1*B*. *D*) *G*_j_ in HeLa cells expressing Cx43m257 (*n* = 3) or wild-type Cx43 (*n* = 12) during superfusion with 40 mM TMA. *E*) Cartoon representation of Cx43m257 protein, highlighting the lack of C tail (left). Resting pH_i_ was preadjusted to acidic level before H^+^ uncaging by 30 mM NH_4_Cl prepulse returned to normal Tyrode that contained 30 µM dimethylamiloride to inhibit pH_i_ recovery (middle). Resting pH_i_ was preadjusted to the alkaline level before H^+^ uncaging by 80 mM acetate prepulse returned to normal Tyrode pH 8.4 to slow pH_i_ recovery (right). *F*) Transmission of acid indicates the persistence of Cx43m257 permeation during acidosis (middle) or alkalosis (right) at levels that were comparable to wild-type Cx43 near resting pH_i_ (left). *G*) Single channel events recorded from N2a-Cx43m257 cells in 2 mM halothane show open conductance that is similar to wild-type channels. *H*) *P*_H_^app^ data compiled from all experiments indicate a lack of sensitivity to acidosis or alkalosis of the tailless mutant (from left to right: *n* = 11, 5, 13). ^#^Significantly different from 0.

Earlier studies have implicated the C tail of the Cx43 protein in the response to low pH_i_ (21, 22). To investigate whether this Cx43 domain is also responsible for alkaline gating, a His-tagged Cx43m257 truncated mutant, referred to as tailless, was transfected into N2a cells (7). Immunostaining using His-tag Abs confirmed the correct targeting to the cell-cell interface (**Fig. 6*A***). The mutant retained its sensitivity to βGA (Fig. 6*B*), but, unlike the full-length construct, cell-to-cell pair conductance demonstrated no response to alkaline pH_i_ (Fig. 6*C, D*). Moreover, acidification or alkalinization of N2a cells that were transfected with the mutant did not inhibit H^+^ ion transmission between coupled cells (Fig. 6*E, F*). In double whole-cell voltage-clamp experiments under superfusion with 20 mM TMA, tailless mutant channel gating did not demonstrate a residual state, but its main conductance state was preserved (Fig. 6*G*), as previously reported at resting pH_i_ (13). In summary, the C tail of Cx43 is involved in mediating both acidic and alkaline gating of assembled Cx43 channels (Fig. 6*H*).

### C tail cysteine residues are implicated in the mechanism of Cx43 channel gating by alkaline pH_i_

**Figure 7.**
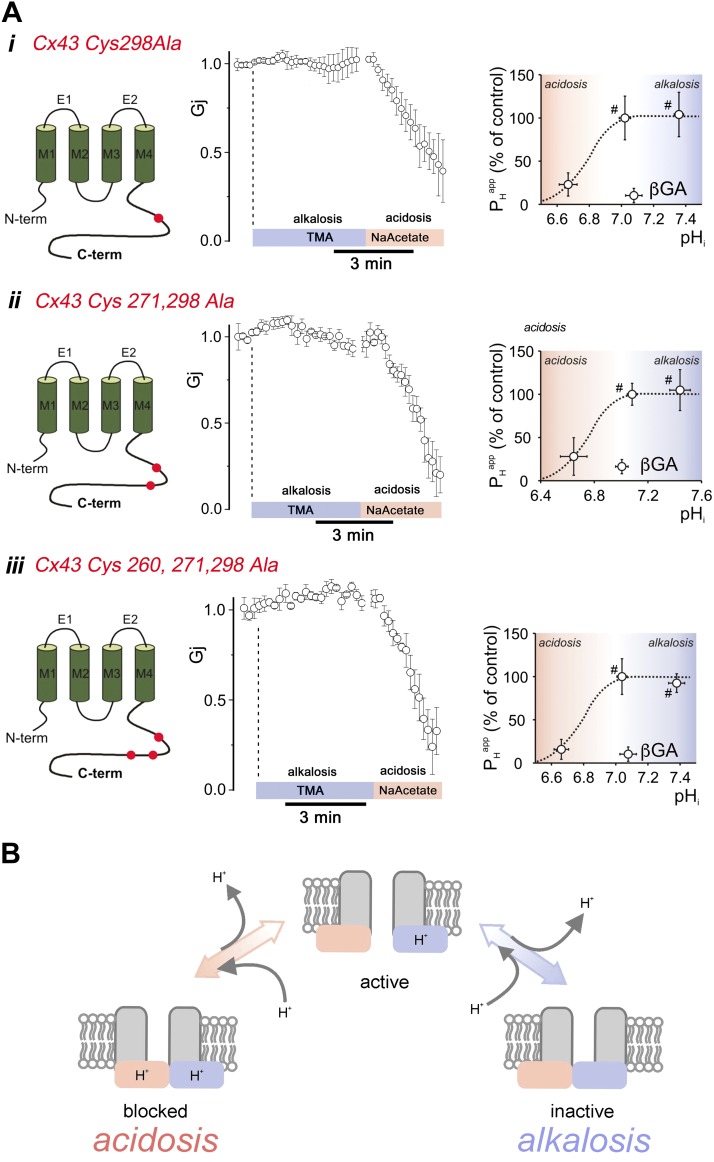
Involvement of cysteine residues in alkali gating. *Ai*) Cysteine 298–alanine Cx43 mutants demonstrated no inhibitory effect of alkalosis on *G*_j_ (40 mM TMA) under double whole-cell voltage clamp in cell triplets (*n* = 4). Eighty millimolar acetate, which induces acidosis, produced a prompt inhibition of mutant Cx43 channel activity (*n* = 3). Effect of high and low pH_i_ on *P*_H_^app^ as determined by H^+^ uncaging showing the ablated response to alkaline pH_i_ but an intact inhibition by acidic pH_i_. *ii*) Similar results were obtained by using Cx43 with substituted cysteine residues 270 and 298 (*n* = 3) and (*iii*) cysteine residues 260, 271, and 298 (*n* = 3). ^#^Significantly different from 0 (*P* < 0.05). *B*) Schematic representation of H^+^ activation and H^+^ inhibition mechanisms on wild-type Cx43 channels. Deprotonated form of Cx43 (lower right) represents the closed state. H^+^ binding to a low-affinity site activates the Cx43 channel. At higher [H^+^], protonation at the inhibitory site, with lower apparent H^+^ affinity, produces H^+^ block. ^#^Significantly different from 0.

Histidine residues of the C tail of Cx43 have been implicated in the gating of channel activity by low pH_i_ (9), which is consistent with the residue’s p*K*_a_ of <7.0. Other residues of the C tail with higher p*K*_a_ are plausible candidates for the alkaline gating. Of these, cysteine, arginine and tyrosine have a p*K*_a_ of >8 and are found in the Cx43 protein (Supplemental Fig. 1). A related connexin isoform, Cx45, which demonstrates greatly attenuated alkaline gating (Supplemental Fig. 7), contains no cysteine in its C tail; thus, these residues are plausible candidates for alkaline gating. To assess whether cysteines that are naturally found in the C tail of Cx43 are linked to alkaline inhibition, residues at positions 298, 271, or 260 were mutated to alanine in various permutations and their effect on *G*_j_ and *P*_H_^app^ was probed in N2a cells. Whereas cysteine, as a free amino acid, has a nominally alkaline p*K*_a_, which renders it suitable as an alkaline sensor, it is important to consider how adjacent residues in a motif affect its ionization state. The 3 residues that flank either side of Cys298 form a NSSCRNY motif, which has an alkaline calculated isoelectric point of p*K*_a_ = 8.5. In contrast, the sequence that flanks Cys260 and Cys271—AKDCGSQ and FNGCSSP, respectively—have ensemble isoelectric points of 6.2 and 5.9, respectively. Thus, Cys260 and Cys271 are less likely to be alkaline sensors because the adjacent, negatively charged glutamate greatly shifts the apparent p*K*_a_ of the motif toward acidic levels. For this reason, a Cys298Ala substitution was first Cx43 mutant constructed. As shown in Fig. 7*A**i*, the Cys298Ala mutant was insensitive to alkaline pH_i_ (TMA superfusion), but retained sensitivity to acidic pH_i_ (acetate superfusion). To investigate whether if either of the 2 other Cys residues have a supplementary effect, mutants with additive Cys-to-Ala substitutions were expressed. As shown in Fig. 7*A**ii, iii*, these additional Cys substitutions had no additional effect on alkaline or acidic responses, which argues that Cys298 is likely to be the principal alkaline-sensing residue in Cx43 protein. Overall, these results can be interpreted in terms of a 2-site model: an inhibitory domain that, upon protonation, leads to channel closure over the acidic pH_i_ range, and an activatory domain that, upon protonation, leads to channel opening over the alkaline pH_i_ range (Fig. 7*B*).

## DISCUSSION

The results of this study demonstrate that Cx43 channels, transfected into HeLa or N2a cells, replicate the pH_i_-dependent gating behavior that has been previously described in cardiac myocytes (12), a type of cell with naturally high Cx43 expression. Thus, heterologously expressed Cx43 channels are a good model with which to study the gating mechanism of Cx43 channels by H^+^ ions. In addition, the similarity in biophysical behavior argues that the biphasic pH_i_ dependence of cell-to-cell coupling demonstrated in cardiac myocytes is not a function of cellular context or molecular components that are unique to the myocardium, but, rather, an intrinsic property of the Cx43 protein. The biophysical properties of heterologously expressed Cx43 channels were interrogated electrophysiologically by measuring junctional conductance and imaging H^+^ ion transmission to quantify a permeability constant. The former, double patch-clamp technique is a validated and robust method for determining connexin channel gating in response to various biophysical or chemical agents (13). In contrast, H^+^ ion transmission—assessed by *P*_H_^app^—is a more recently developed approach (15) that probes connexin activity by using an independent readout and has the advantage of measuring coupling in intact cells that are not impaled by electrodes—for example, without the potential problem of intracellular dialysis.

To map the pH_i_ dependence of Cx43 function, this dual measurement approach was applied for a range of pH_i_ levels that were manipulated by using weak acids or bases. Previously, changes in pH_i_ have typically been induced by altering CO_2_ partial pressure (22), adjusting extracellular pH, or inducing either quasi steady-state pH shifts (21) or shifts of >1 pH unit (7, 9, 23). The major disadvantages of the above-mentioned approaches are the simultaneous modification of pH on either side of the cell membrane and the longer times that are necessary for attaining a target pH_i_ change. These factors make it difficult to disentangle the effects on intra- and extracellular pH. Although some early reports have claimed that coupling is insensitive to external acidification (24), increased extracellular pH is now recognized to play a role in regulating Cx function by favoring disulfide bridge formation and Cx docking (25). This effect becomes more relevant in longer-lasting experiments during which pH_i_ changes are induced slowly and within the time frame of Cx43 protein turnover (10). Our approach to clamping pH_i_ involved the addition—or addition followed by washout—of weak acids–bases. In their uncharged forms, these molecules permeate the cell where they dissociate or combine with H^+^ ions, thereby changing pH_i_ without altering extracellular pH. In our protocols, pH_i_ changes were induced in a matter of seconds, and their effects on Cx43 channel gating were examined simultaneously or within a few minutes.

By demonstrating a biphasic pH_i_ sensitivity of Cx43 function (Fig. 4*E*), the results of this study add to the arguments against the canonical sigmoidal pH_i_ dependence (21). Although it is widely recognized that profound intracellular acidification closes Cx43 channels (7, 9, 21), less is known about the effect of increasing pH_i_. Apart from our earlier study that described a dual response of the ventricular gap junction to H^+^ ions (12) as well as some evidence in the literature of an alkali block of Cx43 channels (21), only 2 contradictory observations have been made for Cx43 channel activity at increased pH_i_. One of these reported an 85% *G*_j_ decrease at pH_i_ 8.1 in Novikoff cells that endogenously expressed Cx43 (26), whereas the other describes a 10% *G*_j_ increase at high pH_i_ for Cx43 expressed in *Xenopus* oocytes (23). The reasons for the discrepancy between our work and the latter study are not clear, although the slow induction of alkalinization and high extracellular pH may be relevant. In addition, *Xenopus* oocytes express Cx38 channels (27) that may contribute to the alkaline-induced increase in coupling.

A noteworthy feature of the *G*_j_ response to pH_i_ is its significant time delay (∼10 or more), as shown in Fig. 1*C, F*. This delay was also reported in earlier work (21, 28, 29) and may suggest a pH_i_-evoked cooperative conformational change that involves all 6 monomers of a Cx channel or the participation of accessory diffusible molecules that are also present in expression systems (30). The time scale of conductance responses cannot, *per se*, exclude a role for protein internalization in the underlying mechanism. Although fast internalization (seconds) has been described for some membrane proteins, such as synaptic vesicle proteins (31), the internalization of gap junction plaques involves a more complicated process (32). Previous studies have demonstrated that Cx43 internalization can be induced during a 30-min period of acute ischemia (33), but, paradoxically, this occurred with no apparent change in junctional conductance because of a compensatory delivery of new hemichannels to the junctional plaque (34, 35). Moreover, the process of reinsertion—that is, reversal of internalization—is expected to be slower because it has to allow sufficient time for the proper docking of juxtaposed hemichannels. Cx43 recycling has been reported to last 30 min (36), have a half-life of ∼2 h in cultured cardiomyocytes (10, 37), or require ∼2 h to complete during mitosis (38). In contrast, our data (Fig. 1) show a similar response time for inhibition—TMA or acetate—and subsequent recovery (removal of TMA/acetate), which is not consistent with the time scale of Cx43 internalization and reinsertion. Together with evidence for complete and fast reversibility, our findings point toward a gating mechanism.

Our results provide insight into the molecular mechanism that underpins the biphasic effect of H^+^ ions on Cx43 channels. The absence of an apparent effect of pH_i_ changes on single-channel conductance (Fig. 2)—also shown previously by others (39)—argues against a pH-dependent alteration in the assembly of Cxs around the central pore, one possible form of gating. The small shift toward 60 pS in the single-channel event distribution during alkalosis may suggest an effect of an altered phosphorylation state; however, experimental data presented in Fig. 7 on the alkaline response of phospho-null Cx43 mutants argue against this. In these experiments, HeLa cells expressed mutant Cx43 with all serine residues [Cx43(S-A)6] of the PKC epitope site substituted for alanine—from 364 to 373, including Ser368, which is responsible for the unitary conductance shift by phosphorylation. These mutants reached levels of *G*_j_ uncoupling and H^+^ flux that were similar to those of wild-type Cx43, which indicated that changes in phosphorylation state cannot explain the response to alkaline or acidic pH_i_. Moreover, Western blots presented in Supplemental Fig. 7 indicate that the ratio between phosphorylated and dephosphorylated Cx43 remains unchanged after a period of intracellular alkalosis or acidosis. The persistence of biphasic pH_i_ gating after loading cells with the Ca^2+^ buffer, BAPTA, argues against the involvement of acid-evoked [Ca^2+^] signals as intermediates of gating (40). Of note, at least a partial involvement of Ca^2+^ ions in the acid-gating mechanism has been proposed to take place in cardiac myocytes and Novikoff hepatoma cell pairs (12, 41), yet this may be a function of cellular context. Previous studies have indicated that changes in Cx43 phosphorylation state at serine residues of the C terminus can influence Cx assembly, their gating, and half-life (42); however, our results demonstrate that a 5-min treatment of Cx43-transfected N2a cells with 80 mM Na^+^ acetate or 30 mM NH_4_Cl did not affect the migration pattern of Cx43 immunoreactivity, which argues against any major shift in phosphorylation state (Supplemental Fig. 7), although certain single-residue changes in Cx43 phosphorylation, such as at Ser368, can occur without detectable migration shifts (42). However, a post-translational change at Ser368 would become apparent from a reduction in unitary conductance (43), which was not observed in our recordings at acid or alkali pH_i_ (Fig. 2). In summary, Ca^2+^ signals and phosphorylation state do not seem to play a role in rapid, pH_i_-induced gating behavior. Instead, the gating mechanism is likely to be an inherent property of the Cx43 protein, which is recapitulated when the channel is assembled in expression systems.

Our measurements on cells that were transfected with Cx43 mutants suggest that C-tail cysteine residues are involved with the mechanism of alkaline gating. First, a tailless Cx43 mutant loses its response to alkaline pH. This observation resembles the behavior of the ball-and-chain inactivation that has been observed in many types of ion channel. Second, substituting cysteine, a residue with an alkaline p*K*_a_, with nontitratable alanine attenuates alkaline gating. This is the first report, to our knowledge, to implicate these C-tail cysteines in Cx channel gating by pH_i_, although residues, such as Cys271, have been linked to redox responses (44) and *S*-nitrosylation by nitric oxide (45). Third, channels formed of Cx45, an isoform that is related to Cx43 but that lacks these critical cysteine residues, demonstrates an attenuated alkaline response compared with Cx43 channels (Supplemental Fig. 6).

The C-tail domain of Cx43 is also responsible for the inhibitory effect of H^+^ ions, but here, the residue that underlies the inhibitory effect is a histidine that is located at the intracellular loop, with an accordingly lower p*K*_a_ (9, 21, 22). The structural basis for the inhibition involves a stabilizing effect of histidine protonation on the α-helical order of the cytoplasmic loop that then favors intramolecular interactions (9, 46).

The superimposition of an activatory effect of H^+^ ions—titrated from an alkaline pH_i_—and an independent inhibitory effect—occurring at more acid pH_i_—produces an overall pH_i_ range that permits physiologic cell-to-cell communication. The finding that peak Cx43 channel function occurs in the range of 6.9–7.0 pH_i_ (Fig. 4*E*) indicates that modest acidification from a resting value of ∼7.2 will strengthen cell-to-cell coupling. This response would favor syncytial dissipation of any pH_i_ gradients—thereby unifying pH_i_-dependent processes—and facilitate tissue functions that rely on cell-to-cell communication, such as the transmission of electrical signals in the heart. The basis for this response is explained by H^+^ activation, which underlies the alkaline range of the biphasic pH_i_ sensitivity of the Cx43 channel. A more profound acidification leads to uncoupling, which can be interpreted as a protective strategy to mitigate for the potential consequences of toxic levels of acid spillover into neighboring cells. In the heart and brain, for instance, this mechanism would help protect cells around ischemic areas and reduce tissue damage propagation (2, 3).

In summary, we demonstrate the phenomena of H^+^ activation and H^+^ inhibition of Cx43 channels as independent channel gating mechanisms. The ensemble of H^+^ activation and inhibition produces a biphasic pH_i_ dependence of Cx43 channel function. This bell-shaped pH_i_ sensitivity ensures that gap junctions are responsive to changes in cytoplasmic acid–base chemistry, which produces a physiologic range of pH_i_ that permits intercellular communication in tissues, such as the heart and brain.

## Supplementary Material

This article includes supplemental data. Please visit *http://www.fasebj.org* to obtain this information.

## Abbreviations

βGA: β-glycyrrhetinic acid
AM: acetoxymethyl ester
Cx: connexin
NHE: Na^+^/H^+^ exchanger
*P*_H_^app^: apparent H^+^ ion permeability constant
pH_i_: intracellular pH
SNARF-1: seminaphtharhodafluor-1
TMA: trimethylamine

## References

[1] Kumar N. M., Gilula N. B. (1996). The gap junction communication channel.. Cell.

[2] Lin J. H. C., Weigel H., Cotrina M. L., Liu S., Bueno E., Hansen A. J., Hansen T. W., Goldman S., Nedergaard M. (1998). Gap-junction-mediated propagation and amplification of cell injury.. Nat. Neurosci..

[3] Peters N. S., Coromilas J., Severs N. J., Wit A. L. (1997). Disturbed connexin43 gap junction distribution correlates with the location of reentrant circuits in the epicardial border zone of healing canine infarcts that cause ventricular tachycardia.. Circulation.

[4] Vaughan-Jones R. D., Spitzer K. W., Swietach P. (2009). Intracellular pH regulation in heart.. J. Mol. Cell. Cardiol..

[5] Noma A., Tsuboi N. (1987). Dependence of junctional conductance on proton, calcium and magnesium ions in cardiac paired cells of guinea-pig.. J. Physiol..

[6] Spray D. C., Harris A. L., Bennett M. V. (1981). Gap junctional conductance is a simple and sensitive function of intracellular pH.. Science.

[7] Ek-Vitorín J. F., Calero G., Morley G. E., Coombs W., Taffet S. M., Delmar M. (1996). pH regulation of connexin43: molecular analysis of the gating particle.. Biophys. J..

[8] Duffy H. S., Sorgen P. L., Girvin M. E., O’Donnell P., Coombs W., Taffet S. M., Delmar M., Spray D. C. (2002). pH-dependent intramolecular binding and structure involving Cx43 cytoplasmic domains.. J. Biol. Chem..

[9] Ek J. F., Delmar M., Perzova R., Taffet S. M. (1994). Role of histidine 95 on pH gating of the cardiac gap junction protein connexin43.. Circ. Res..

[10] Laird D. W., Puranam K. L., Revel J. P. (1991). Turnover and phosphorylation dynamics of connexin43 gap junction protein in cultured cardiac myocytes.. Biochem. J..

[11] Elias L. A., Wang D. D., Kriegstein A. R. (2007). Gap junction adhesion is necessary for radial migration in the neocortex.. Nature.

[12] Swietach P., Rossini A., Spitzer K. W., Vaughan-Jones R. D. (2007). H^+^ ion activation and inactivation of the ventricular gap junction: a basis for spatial regulation of intracellular pH.. Circ. Res..

[13] Moreno A. P., Chanson M., Elenes S., Anumonwo J., Scerri I., Gu H., Taffet S. M., Delmar M. (2002). Role of the carboxyl terminal of connexin43 in transjunctional fast voltage gating.. Circ. Res..

[14] Leem C. H., Lagadic-Gossmann D., Vaughan-Jones R. D. (1999). Characterization of intracellular pH regulation in the guinea-pig ventricular myocyte.. J. Physiol..

[15] Swietach P., Spitzer K. W., Vaughan-Jones R. D. (2007). pH-dependence of extrinsic and intrinsic H^+^-ion mobility in the rat ventricular myocyte, investigated using flash photolysis of a caged-H^+^ compound.. Biophys. J..

[16] Dovmark T. H., Saccomano M., Hulikova A., Alves F., Swietach P. (2017). Connexin-43 channels are a pathway for discharging lactate from glycolytic pancreatic ductal adenocarcinoma cells.. Oncogene.

[17] Zaniboni M., Rossini A., Swietach P., Banger N., Spitzer K. W., Vaughan-Jones R. D. (2003). Proton permeation through the myocardial gap junction.. Circ. Res..

[18] Swietach P., Vaughan-Jones R. D. (2005). Relationship between intracellular pH and proton mobility in rat and guinea-pig ventricular myocytes.. J. Physiol..

[19] Swietach P., Youm J.-B., Saegusa N., Leem C.-H., Spitzer K. W., Vaughan-Jones R. D. (2013). Coupled Ca^2+^/H^+^ transport by cytoplasmic buffers regulates local Ca^2+^ and H^+^ ion signaling.. Proc. Natl. Acad. Sci. USA.

[20] Lurtz M. M., Louis C. F. (2007). Intracellular calcium regulation of connexin43.. Am. J. Physiol. Cell Physiol..

[21] Liu S., Taffet S., Stoner L., Delmar M., Vallano M. L., Jalife J. (1993). A structural basis for the unequal sensitivity of the major cardiac and liver gap junctions to intracellular acidification: the carboxyl tail length.. Biophys. J..

[22] Morley G. E., Taffet S. M., Delmar M. (1996). Intramolecular interactions mediate pH regulation of connexin43 channels.. Biophys. J..

[23] González-Nieto D., Gómez-Hernández J. M., Larrosa B., Gutiérrez C., Muñoz M. D., Fasciani I., O’Brien J., Zappalà A., Cicirata F., Barrio L. C. (2008). Regulation of neuronal connexin-36 channels by pH.. Proc. Natl. Acad. Sci. USA.

[24] Turin L., Warner A. (1977). Carbon dioxide reversibly abolishes ionic communication between cells of early amphibian embryo.. Nature.

[25] Dahl G., Levine E., Rabadan-Diehl C., Werner R. (1991). Cell/cell channel formation involves disulfide exchange.. Eur. J. Biochem..

[26] Skeberdis V. A., Rimkute L., Skeberdyte A., Paulauskas N., Bukauskas F. F. (2011). pH-dependent modulation of connexin-based gap junctional uncouplers.. J. Physiol..

[27] Gimlich R. L., Kumar N. M., Gilula N. B. (1988). Sequence and developmental expression of mRNA coding for a gap junction protein in *Xenopus*.. J. Cell Biol..

[28] Palacios-Prado N., Sonntag S., Skeberdis V. A., Willecke K., Bukauskas F. F. (2009). Gating, permselectivity and pH-dependent modulation of channels formed by connexin57, a major connexin of horizontal cells in the mouse retina.. J. Physiol..

[29] Palacios-Prado N., Briggs S. W., Skeberdis V. A., Pranevicius M., Bennett M. V. L., Bukauskas F. F. (2010). pH-dependent modulation of voltage gating in connexin45 homotypic and connexin45/connexin43 heterotypic gap junctions.. Proc. Natl. Acad. Sci. USA.

[30] Trexler E. B., Bukauskas F. F., Bennett M. V., Bargiello T. A., Verselis V. K. (1999). Rapid and direct effects of pH on connexins revealed by the connexin46 hemichannel preparation.. J. Gen. Physiol..

[31] Hull C., von Gersdorff H. (2004). Fast endocytosis is inhibited by GABA-mediated chloride influx at a presynaptic terminal.. Neuron.

[32] Thévenin A. F., Kowal T. J., Fong J. T., Kells R. M., Fisher C. G., Falk M. M. (2013). Proteins and mechanisms regulating gap-junction assembly, internalization, and degradation.. Physiology (Bethesda).

[33] Smyth J. W., Zhang S.-S., Sanchez J. M., Lamouille S., Vogan J. M., Hesketh G. G., Hong T., Tomaselli G. F., Shaw R. M. (2014). A 14-3-3 mode-1 binding motif initiates gap junction internalization during acute cardiac ischemia.. Traffic.

[34] Xie H., Cui Y., Hou S., Wang J., Miao J., Deng F., Feng J. (2017). Evaluation of connexin 43 redistribution and endocytosis in astrocytes subjected to ischemia/reperfusion or oxygen-glucose deprivation and reoxygenation.. BioMed Res. Int..

[35] Gaietta G., Deerinck T. J., Adams S. R., Bouwer J., Tour O., Laird D. W., Sosinsky G. E., Tsien R. Y., Ellisman M. H. (2002). Multicolor and electron microscopic imaging of connexin trafficking.. Science.

[36] Gilleron J., Carette D., Fiorini C., Dompierre J., Macia E., Denizot J.-P., Segretain D., Pointis G. (2011). The large GTPase dynamin2: a new player in connexin 43 gap junction endocytosis, recycling and degradation.. Int. J. Biochem. Cell Biol..

[37] Darrow B. J., Laing J. G., Lampe P. D., Saffitz J. E., Beyer E. C. (1995). Expression of multiple connexins in cultured neonatal rat ventricular myocytes.. Circ. Res..

[38] Boassa D., Solan J. L., Papas A., Thornton P., Lampe P. D., Sosinsky G. E. (2010). Trafficking and recycling of the connexin43 gap junction protein during mitosis.. Traffic.

[39] Bukauskas F. F., Bukauskiene A., Bennett M. V., Verselis V. K. (2001). Gating properties of gap junction channels assembled from connexin43 and connexin43 fused with green fluorescent protein.. Biophys. J..

[40] Willoughby D., Thomas R., Schwiening C. (2001). The effects of intracellular pH changes on resting cytosolic calcium in voltage-clamped snail neurones.. J. Physiol..

[41] Lazrak A., Peracchia C. (1993). Gap junction gating sensitivity to physiological internal calcium regardless of pH in Novikoff hepatoma cells.. Biophys. J..

[42] Solan J. L., Lampe P. D. (2009). Connexin43 phosphorylation: structural changes and biological effects.. Biochem. J..

[43] Lampe P. D., TenBroek E. M., Burt J. M., Kurata W. E., Johnson R. G., Lau A. F. (2000). Phosphorylation of connexin43 on serine368 by protein kinase C regulates gap junctional communication.. J. Cell Biol..

[44] Pogoda K., Kameritsch P., Retamal M. A., Vega J. L. (2016) Regulation of gap junction channels and hemichannels by phosphorylation and redox changes: a revision. BMC Cell Biol. 17 (Suppl 1), 11 10.1186/s12860-016-0099-3 27229925 PMC4896245

[45] Straub A. C., Billaud M., Johnstone S. R., Best A. K., Yemen S., Dwyer S. T., Looft-Wilson R., Lysiak J. J., Gaston B., Palmer L., Isakson B. E. (2011). Compartmentalized connexin 43 s-nitrosylation/denitrosylation regulates heterocellular communication in the vessel wall.. Arterioscler. Thromb. Vasc. Biol..

[46] Sorgen P. L., Duffy H. S., Cahill S. M., Coombs W., Spray D. C., Delmar M., Girvin M. E. (2002). Sequence-specific resonance assignment of the carboxyl terminal domain of connexin43.. J. Biomol. NMR.

