# Supplementary material for: Distinct moieties underlie biphasic H+ gating of connexin43 channels, producing a pH optimum for intercellular communication
Source: FASEB J. 2018 Jan 5;32(4):1969–81. doi: 10.1096/fj.201700876R (PMC5893178; doi:10.1096/fj.201700876R)
Supplement: Supplementary file 1 [file fj.201700876R.sf1.docx]

**Supplementary Figures**


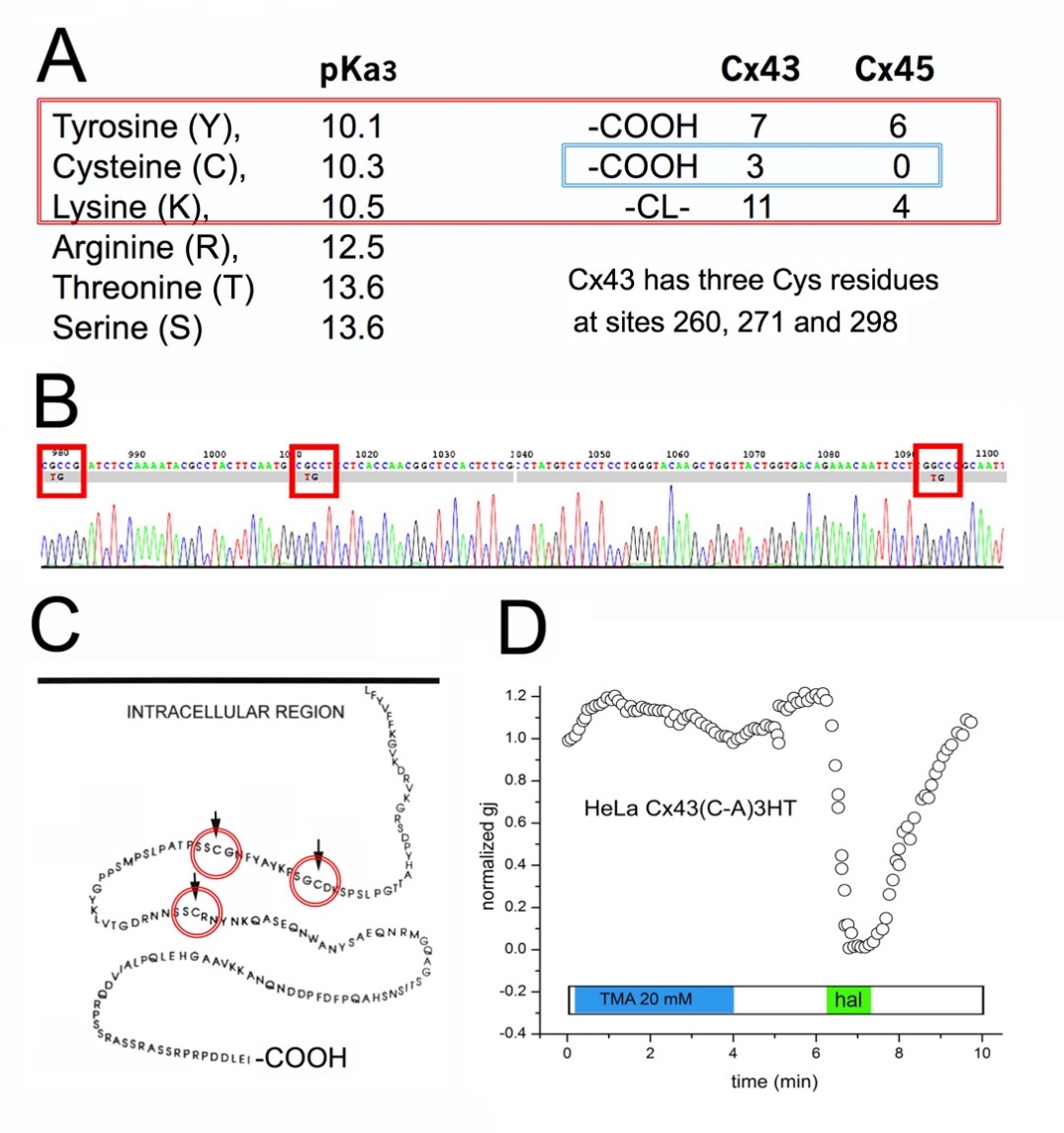
**Figure S1**. **(A)** Amino acid residues present in Cx43 and Cx45 of pK_a_ in the alkaline range (sorted by third dissociation constant, pK_a3_). Values for Tyrosine, Cysteine and Lysine (red box) are nearest to physiological pH. Cysteine residues are present in Cx43 and not Cx45, which correlates with sensitivity to alkalosis observed only in the former. Thus, C-terminus cysteines were selected for mutagenesis. **(B)** DNA sequence and their corresponding chromatogram peaks indicating the sites (red squares) where mutagenesis was applied to substitute cysteine residues with alanine residues. **(C)** Amino acid sequence of the intracellular C-tail of Cx43, where the cysteine residues are indicated (red circle). **(D)** Gating of mutant Cx43 channels expressed in N2a cells in which all cysteine residues were substituted by alanine, showing response to 20 mM TMA and 5 mM halothane. The response to TMA was abolished but halothane closes the channels.


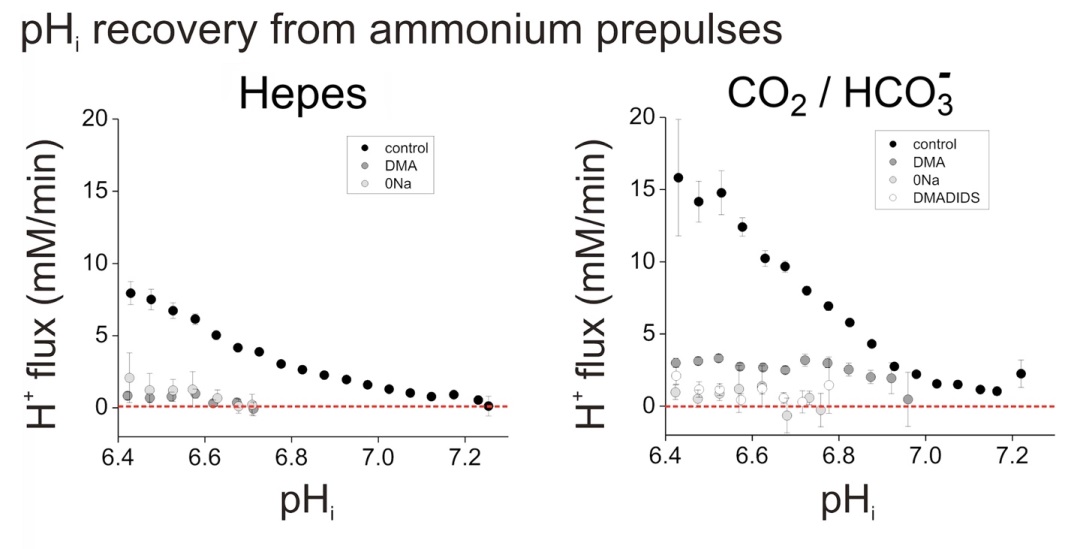
**Figure S2.** pH-dependence of acid extrusion flux measured in HeLaCx43 cells. Transmembrane H^+^/H^+^-equivalent fluxes in **(A)** Hepes or **(B)** CO_2_/HCO_3_^-^ buffered superfusates. Flux inhibitable by 30 μM dimethylamiloride (DMA) or removal of extracellular Na^+^ is attributable to NHE. In CO_2_/HCO_3_^-^, flux was ablated by 30 μM DMA plus 150 μM 4,4′-diisothio-cyanostilbene 2,2′-disulphonic acid (DIDS). The DIDS-inhibitable component of flux in CO_2_/HCO_3_^-^ buffer is attributable to Na^+^-HCO_3_^-^ cotransporters (NBC).


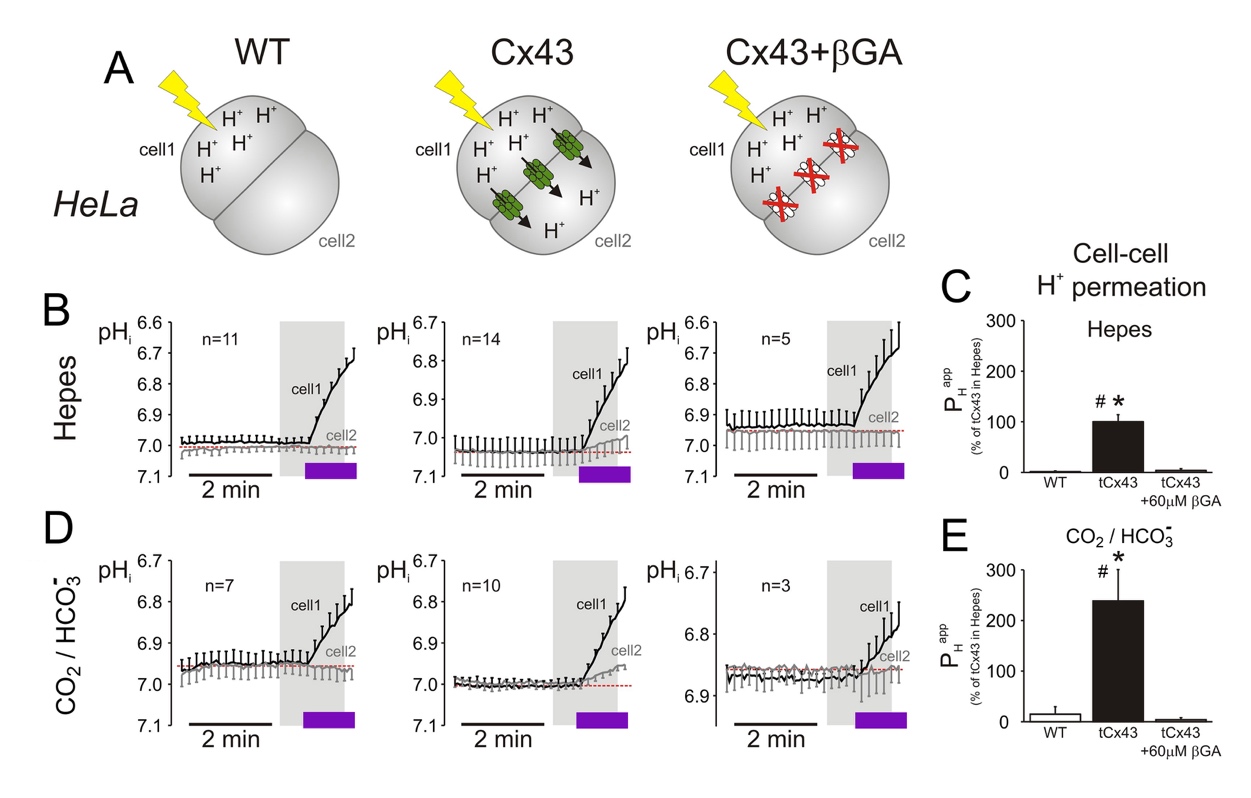
**Figure S3.** Calculating junctional H^+^ ion permeation in HeLa cell-pairs positive or negative for Cx43 expression. **(A).** H^+^ were photolytically uncaged (triple-flash of UV light every 3.6 sec in 5μm x 5μm ROI) within cell-1 from intracellular 2-nitrobenzaldehyde (1mM). pH_i_ was imaged confocally (cSNARF-1) in both cells of the pair to determine junctional H^+^ ion permeation. **(B).** Significant H^+^ ion permeation was observed between Cx43-expressing Hela cells (central panel), and this was sensitive to 60 μM β-glycyrrhetinic acid (right panel), but was absent in wild-type cells lacking Cx43 (left panel). Measurements in Hepes-buffered solution containing 30 μM dimethylamiloride (DMA). To better illustrate the pH_i_ changes in cell-2, a dashed line was drawn to extrapolate baseline changes. **(C).** P_H_^app^ was calculated for the three conditions. **(D).** Similar experiments were performed for the three experimental conditions illustrated in A in CO_2_/HCO_3_^-^ buffered solution with 30 μM DMA and 150 µM DIDS. **(E).** P_H_^app^ was calculated for the three conditions. * P<0.001.


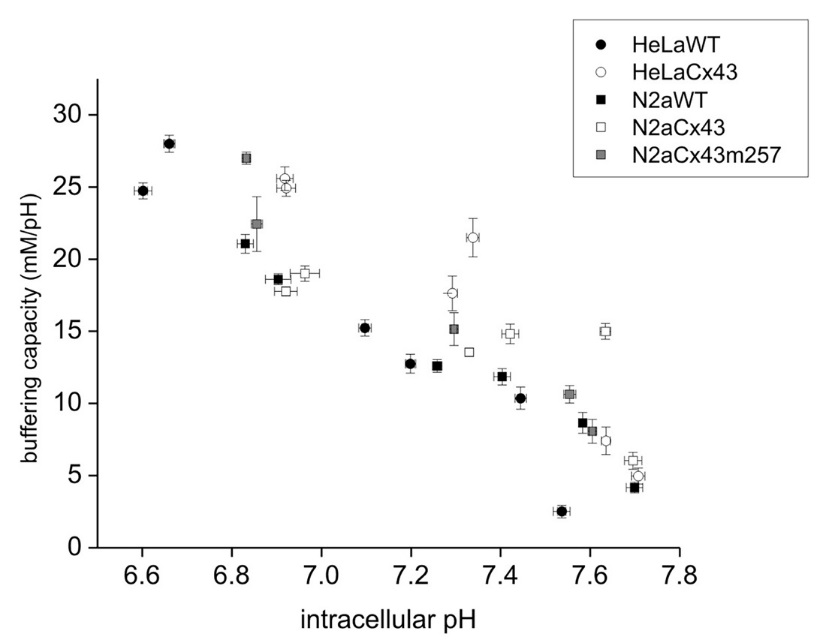


**Figure S4.** Plot of the intrinsic buffering capacity versus pH_i_ measured for all cellular types and expression systems used in our experiments. Method based on Leem, C. H., Lagadic-Gossmann, D., and Vaughan-Jones, R. D. (1999) Characterization of intracellular pH regulation in the guinea-pig ventricular myocyte. The Journal of Physiology 517, 159-180.


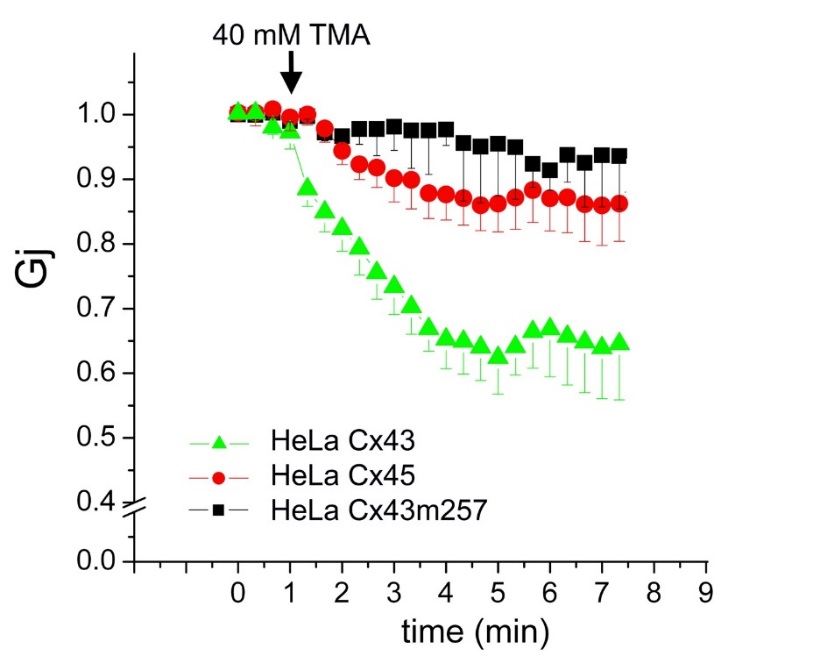

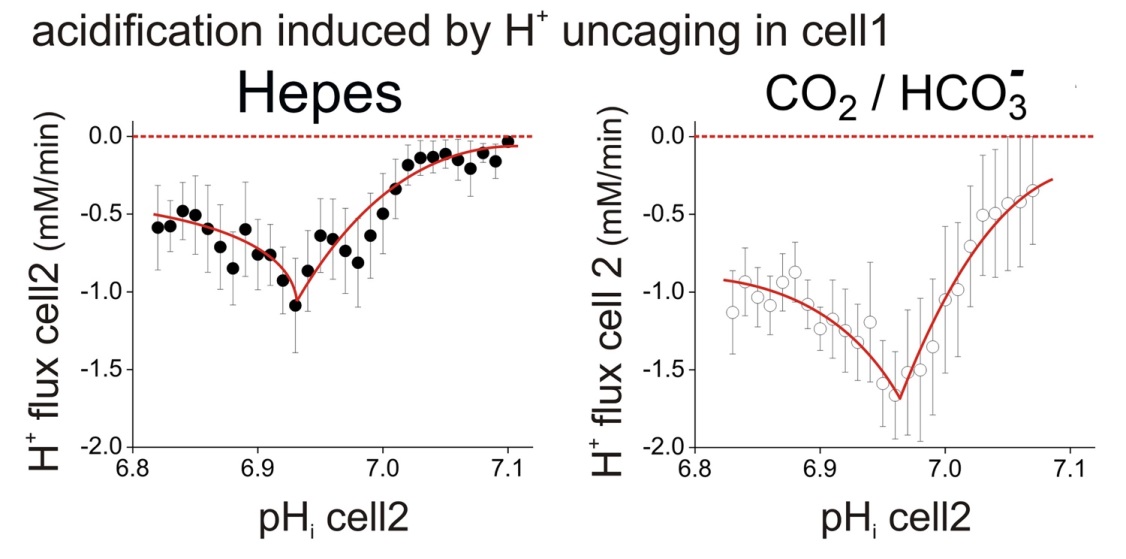
**Figure S5.** Transmitted H^+^ flux (mM/min) into cell-2, as a function of pH_i_ during H^+^ uncaging in cell-1. Experiments performed on Cx43-expressing HeLa cell-pairs. Fluxes were determined as product of buffering capacity (β) and rate of pH_i_ change (dpH_i_/dt). β in cell-2 was calculated from experimental values of buffering capacity (Figure S4) and dpH_i_/dt in cell-2 was taken from Figure 3 D and E, middle panels. H^+^ flux into cell-2 was maximal at pH_i_ 6.9-7.0 for both extracellular buffers.

**Figure S6.** Gap junctional coupling between wild-type Cx43, Cx45 and truncated Cx43m257 connexins expressed in HeLa cells in response to 40 mM TMA. Cx45 response (red, n=3)) is less than 50% of that of wild type Cx43 (green, n=12). Cx43m257 (without carboxyl terminal domain) is largely insensitive to changes in pH_i_ induced by TMA (black, n=4)


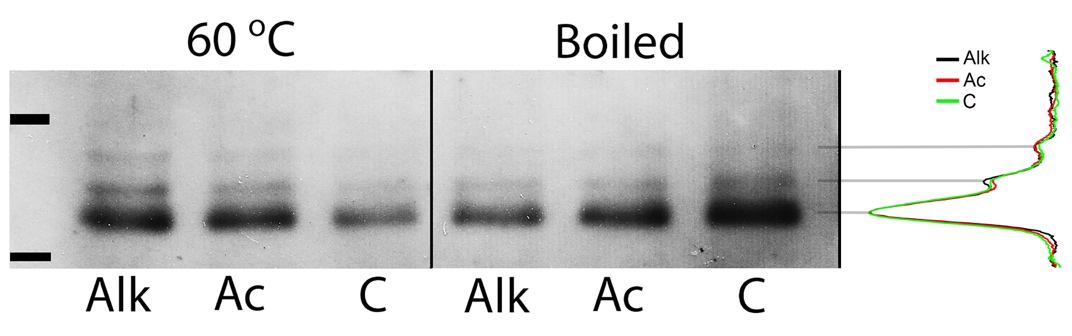
**Figure S7.** Cx43-transfected N2a cells. Changes imposed on pH_i_ did not alter the distribution of Cx43 immunoreactivity bands corresponding to various phosphorylation states. Confluent monolayers of N2a cells, grown in 6cm diameter Petri dishes, were exposed to 5-minute acidosis (80 mM Na^+^-acetate + 30μM DMA) or alkalosis (40 mM TMA). Unstimulated monolayers were used as control. Total protein (see Methods) and samples were either incubated at 60^o^C or boiled for 5 minutes, then subjected to SDS-PAGE and probed with a rabbit polyclonal antibody followed by secondary antibody coupled to horseradish peroxidase. Cx43-corresponding signal was detected by chemiluminescence. Markers on the left correspond to MW 50 kDa (top) and 37 kDa (bottom). Neither acidosis nor alkalosis altered the phosphorylation status of Cx43, as determined by the pixel intensity analysis of the three pH conditions, shown on plot *(right)*. Intensity was determined by averaging signal within each lane, offsetting by the background determined as an interpolation of signal below 35 kDa and above 65 kDa, and normalizing to the peak.
